# Evaluation of IL-17 and IL-35 in patients with giardiasis in Thi-Qar province, Iraq

**DOI:** 10.25122/jml-2021-0328

**Published:** 2022-09

**Authors:** Wed Shakir Hadi, Rabab Shaker Salman, Ali Abdulzahra Al-Fahham, Muhammad Usman Faryad Khan, Sunarto Kadir, Methaq Hadi Laft, Balsam Qubais Saeed, Wesam Radhi Kadhum, Abduladheem Turki Jalil, Mustafa Mohammed Kadhim

**Affiliations:** 1 Directorate of Dhi Qar Education, Ministry of Education, Dhi Qar, Iraq; 2 Department of Physiology, Pharmacology and Chemistry, College of Veterinary Medicine, Thi Qar University, Dhi Qar, Iraq; 3 Basic Science Department, Faculty of Nursing, University of Kufa, Kufa, Iraq; 4 State Key Laboratory of Silkworm Genome Biology, Southwest University, Chongqing, China; 5 Faculty of Sports and Health Sciences, Universitas Negeri Gorontalo, Gorontalo, Indonesia; 6 Iraqi Ministry of Education, Al-Qadisiyah, Iraq; 7 Clinical Sciences Department, College of Medicine, University of Sharjah, Sharjah,United Arab Emirates; 8 Department of Pharmacy, Kut College University, Kut, Iraq; 9 Medical Laboratories Techniques Department, Al-Mustaqbal University College, Babylon, Iraq; 10 Department of Dentistry, Kut College University, Kut, Iraq; 11 College of Technical Engineering, The Islamic University, Najaf, Iraq

**Keywords:** IL-17, IL-35, *Giardia lamblia*, giardiasis, diarrhea

## Abstract

*Giardia lamblia, Entamoeba histolytica, Cryptosporidium*, and *Blastocystis* are some parasites primarily responsible for human infections. *Giardia lamblia*, also known as *Giardia intestinalis* or *Giardia duodenalis*, is a common pathogenic protozoan found in the human duodenum and jejunum that causes giardiasis. This study collected stool and blood samples from patients with diarrhea aged less than 1 month to 15 years, from September 2020 to December 2020, in Thi-Qar province. Our study aimed to reveal the diagnosis of *Giardia lamblia* using direct microscopy examination and detect some immunological parameters such as IL-17 and IL-35 in patients infected with giardiasis.

## INTRODUCTION

Diarrhea is characterized as loose, watery stools with a constant need to urinate, commonly described as three or more watery or loose bowel movements each day [1]. Many pathogens cause diarrhea like parasites (*Giardia lamblia, Cryptosporidium parvum, Balantidium coli, E. histolytica*, and *Isosporo billi*.), bacteria (*Aeromonas hydrophila, Clostridium difficile, Helicobacter pylori, Shigella* or *Salmonella* species), and viruses (norovirus and rotavirus), or other unknown reasons [2].

There are many transmission methods to humans, including direct contact with infected people or animals. As for direct contact, a person can get infected by other persons carrying germs via direct contact with blood or body fluids [3].

*Giardia* occurs in two varieties: trophozoites and cysts. The *Giardia lamblia* trophozoite is a heart-shaped organism that measures around 15 meters in length and has four pairs of flagella. A large concave sucking disk on the ventral side helps the organism stick to the intestinal villi.

The *Giardia* forms cysts in the colon, and then the cysts are excreted [4, 5]. They are oval in shape, thick-walled, and very resistant, with a length of 8–14 m with two nuclei in juvenile forms and four nuclei in mature cysts [6–9].

## MATERIAL AND METHODS

### Stool Samples

67 stool samples were obtained from individuals with diarrhea in Thi-Qar province between September and December 2020. The ages of the participants varied from 1 month to 15 years, with 41 men and 26 females. Fresh fecal samples were collected in sterile containers to avoid contamination and stored in hospital laboratories for microscopic inspection [10].

### Blood Samples

Three milliliters of blood samples were collected in a plane tube from 67 patients with diarrhea who were infected with *G. lamblia* and 3 ml of blood samples from 20 healthy volunteer children. The serum was obtained by centrifugation at 3000 rpm and then stored directly at -20°C for immunological ELISA (enzyme-linked immuno-sorbent assay) tests, including IL-17 and IL-35.

### Microscopic Examination

#### Direct wet mount using normal saline 0.9% method

To assess the morphology and mobility of the parasite, the feces sample was emulsified in normal saline. A droplet of normal saline was positioned on the center of the slide, and a slight portion of the stool specimen sample was mixed and emulsified in the saline using a sterile swab stick and examined microscopically using ×10 and ×40 objectives [11–13].

#### Direct smear using Lugol's Iodine

Lugol's iodine is an aqueous solution containing iodine 5% and potassium iodide 10%. The slides were prepared in the same procedures as the direct wet amount except for exchanging the normal saline with Lugol's iodine solution [14].

#### Human Interleukin (IL-17, IL-35) ELISA technique

To detect IL-17 and IL-35 using ELISA test, 67 blood samples were taken from infected patients diagnosed with *G. Lamblia*, and 20 blood samples from healthy male and female children ages less than a year and 15 years as control. Interleukin levels were measured using techniques developed by the company BIO-TEC (Cat.No. E0054Hu, Cat.No.E0042Hu).

### Statistical Analysis

All data were statistically analyzed using Microsoft Excel (version 2010) and SPSS version 24. ANOVA (analysis of variance) was used for multiple independent tests, student t-test for two independent groups, Spearman for correlation, and Chi-square to test the association between two categorical variables [15].

## RESULTS

### Level of IL-17 for patients and controls according to age groups

The results revealed that all groups of patients had statistically significant changes in IL-17 levels when compared to healthy controls (p-value=0.001). High levels of IL-17 were found in the third age group of patients (6–10 years), with a level of 1184.9±272.3 ng/ml, and in the third age group of controls, with a level of 389.49±77.13 ng/ml (Table 1).

**Table 1:** Level of IL-17 for patients and controls according to age groups.

Groups	Age (years)	No. of cases	IL-17 ng/ml M+SD	P-value
**Patient**	<1	15	913.1±106.1	<0.001
**Control**		4	283.7±19.2	
**Patient**	1–5	23	1043.2±205.7	<0.001
**Control**		7	326.4±79.5	
**Patient**	6–10	19	1184.9±272.3	<0.001
**Control**		6	389.49±77.13	
**Patient**	11–15	10	1079.4±55.68	<0.001
**Control**		3	289.3±228.5	
**Total Patients=67; Total Control=20**				

### Level of IL-35 for patients and control according to age groups

The study discovered significant levels of IL-35 in the third age group of patients (375.0±67.7 ng/ml) and high levels in the second age group of controls (8.117±3.2 ng/ml). As shown in Table 2, there were statistically significant variations in IL-35 levels across all patient groups compared to matching healthy control groups (p-value=0.001).

**Table 2:** Level of IL-35 for patient and control according to age groups.

Groups	Age (years)	No. of cases	IL-35 ng/ml M+SD	P-value
**Patient**	<1	15	194.18±9.2	<0.001
**Control**		4	5.13±1.22	
**Patient**	1–5	23	210±24.5	<0.001
**Control**		7	8.317±3.2	
**Patient**	6–10	19	375.0±67.7	<0.001
**Control**		6	6.215±2.55	
**Patient**	11–15	10	310.3±49.7	<0.001
**Control**		3	7.921±3.44	
**Total Patients=67; Total Control=20**				

## DISCUSSION

When we analyzed and compared giardiasis patients with healthy control of all ages, we found high levels of IL-17. This suggests that the concentration of interleukins is not affected by the patient's age but rather by the quantity and concentration of parasites and the patient's immunological condition. This is consistent with Jalil AT et al. (2021) [16], who investigated the role of IL-17 in vaccination-mediated protection and discovered that this cytokine contributed to LecA-alum vaccine protection through neutralization studies. IL-17 cells are recognized to play a significant role in mucosal infection defense. The mechanism of IL-17-mediated protection involves neutrophil recruitment to infected areas and IL-17-induced cytokines and chemokines regulating DC (dendritic cells) activity and Th1 responses [17–23]. The current findings revealed a statistically significant increase in IL-35 levels in patients compared to healthy controls in all age groups. The current study may agree with Cao and his colleagues in Chongqing, China, who discovered that IL-35 levels in blood samples from adult or child patients with sepsis were considerably greater than healthy controls and rose progressively with sepsis severity [24–26]. Similarly, our findings, along with those of Vakili-Samiani et al. (2021) [27] in Mexico City, imply that elevated expression of IL-35 and IL-37 may be responsible for the down-regulation of inflammation in active inflammatory bowel disease (IBD) patients. T cells that release IL-35 and have suppressive activities can be generated in the intestines of mice infected with the intestinal parasite Trichuris muris, according to Choi et al. (2015) [28].

Furthermore, Choi and his colleagues discovered that IL-35 suppresses inflammation by inducing Treg cells and suppressing Th1 and Th17 cells [29–34]. These findings support Dong and Yang's (2015) findings that IL-35 is involved in various chronic inflammatory illnesses and parasitic/bacterial infections when inhibitory cytokines are present [35–40].

## CONCLUSION

The high levels of IL-17 and IL-35 in patients with giardiasis infection indicate the important role of interleukin in activating the immune system during intestinal inflammation.
